# Mn-promoted MoS_2_ catalysts for CO_2_ hydrogenation: enhanced methanol selectivity due to MoS_2_/MnO_*x*_ interfaces†

**DOI:** 10.1039/d3cy01711g

**Published:** 2024-02-02

**Authors:** Gustavo A. S. Alves, Gernot Pacholik, Stephan Pollitt, Tobias Wagner, Raffael Rameshan, Christoph Rameshan, Karin Föttinger

**Affiliations:** a Institute of Materials Chemistry, TU Wien Getreidemarkt 9/BC/01 1060 Vienna Austria karin.foettinger@tuwien.ac.at; b Paul Scherrer Institut (PSI) Forschungsstrasse 111 5232 Villigen Switzerland; c Chair of Physical Chemistry, Montanuniversität Leoben Franz-Josef-Straße 18 8700 Leoben Austria

## Abstract

Considering the alarming scenario of climate change, CO_2_ hydrogenation to methanol is considered a key process for phasing out fossil fuels by means of CO_2_ utilization. In this context, MoS_2_ catalysts have recently shown to be promising catalysts for this reaction, especially in the presence of abundant basal-plane sulfur vacancies and due to synergistic mechanisms with other phases. In this work, Mn-promoted MoS_2_ prepared by a hydrothermal method presents considerable selectivity for CO_2_ hydrogenation to methanol in comparison with pure MoS_2_ and other promoters such as K and Co. Interestingly, if CO is used as a carbon source for the reaction, methanol production is remarkably lower, which suggests the absence of a CO intermediate during CO_2_ hydrogenation to methanol. After optimization of synthesis parameters, a methanol selectivity of 64% is achieved at a CO_2_ conversion of 2.8% under 180 °C. According to material characterization by X-ray Diffraction and X-ray Absorption, the Mn promoter is present mainly in the form of MnO and MnCO_3_ phases, with the latter undergoing convertion to MnO upon H_2_ pretreatment. However, following exposure to reaction conditions, X-ray photoelectron spectroscopy suggests that higher oxidation states of Mn may be present at the surface, suggesting that the improved catalytic activity for CO_2_ hydrogenation to methanol arises from a synergy between MoS_2_ and MnO_*x*_ at the catalyst surface.

## Abstract 2

Considering the alarming scenario of climate change, CO_2_ hydrogenation to methanol is considered a key process for phasing out fossil fuels by means of CO_2_ utilization.

## Graphical abstract

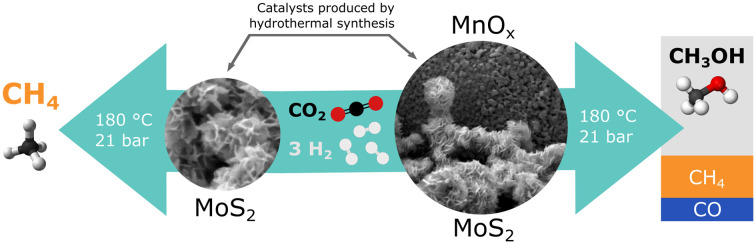

## Introduction

With the alarming scenario of anthropogenic climate change due to persistently high emission levels of CO_2_ from fossil fuels, Carbon Capture and Utilization (CCU) technologies may play a role in the transition of industry towards a net zero future.^1^ Moving away from fossil fuels, CO_2_ could be implemented as an alternative carbon feedstock in a variety of industrial processes.^2,3^ In this context, the production of methanol has been attracting particular interest, given its current role as a critical building block in the chemical industry and the development of sustainable aviation fuel in the near future.^4,5^

Methanol synthesis from CO-rich syngas derived from natural gas using Cu/ZnO/Al_2_O_3_ catalysts has been a well-established industrial process over the past decades. However, replacing fossil resources by alternative carbon sources such as biomass or captured CO_2_ will require the development of new processes, among which the direct CO_2_ hydrogenation may play a decisive role. In comparison with the traditional CO-based route, inherent limitations of the CO_2_ hydrogenation reaction involve its less exothermic character, in addition to the simultaneous production of water.^6^ Nevertheless, reports from the first industrial plant devoted to CO_2_ hydrogenation to methanol suggest significant advantages, being less energy-intensive and producing fewer reaction byproducts.^7^

Similarly as in the conventional methanol synthesis process, copper-based catalysts have been widely regarded as the most effective materials for CO_2_ hydrogenation. Following extensive research, several structural-property relations have been identified for the Cu/ZnO system.^8–10^ Although some copper-based catalysts may produce methanol through the Reverse Water-Gas Shift + CO hydrogenation pathway,^11,12^ the Cu/ZnO interaction has been strongly associated with CO_2_ hydrogenation *via* formate intermediate, according to theoretical studies and operando characterization.^8,13,14^

In addition to such materials, other recent lines of research involve the search for novel catalysts, since the application of copper-based materials is still limited by the instability of the active phase,^15^ low selectivity to methanol^16^ and surface poisoning in the presence of sulfur-containing gases.^17^ In virtue of these issues, non-metallic catalysts such as ZnZrO*x* (ref. 18) and In_2_O_3_ (ref. 19) have attracted growing interest as potential alternatives for this application due to their high stability and selectivity towards methanol.

More recently, similarly selective CO_2_ hydrogenation to methanol has also been demonstrated using MoS_2_ catalysts. In contrast with the aforementioned materials which optimally operate above 250 °C, MoS_2_ can be active at remarkably low temperatures around 180 °C.^20^ In addition to the more favorable thermodynamics for methanol formation, these mild operation conditions could offer an economical advantage with respect to other catalysts, in the context of large-scale applications. However, CO_2_ hydrogenation to methanol can only be achieved in MoS_2_ with specific properties, as under the typical reaction conditions most formulations of pure MoS_2_ promote the formation of methane instead of methanol. In fact, only in the presence of basal plane S-vacancies CO_2_ was shown to dissociate to CO and O at low temperatures, thus allowing for selective methanol production without further hydrogenation to methane.^20^ This suggests that in favor of selective CO_2_ hydrogenation to methanol, basal-plane sulfur vacancies should be obtained by preparation methods that induce the formation of sheet-like structures instead of edge-rich nanoparticles.

Moreover, in addition to the MoS_2_ morphology, methanol selectivity can arise due to other effects. In a recent study, a MoS_2_/ZnS catalyst produced by a solvothermal route involving a metal–organic framework precursor has shown high selectivity to methanol, as a possible result of ZnS blocking MoS_2_ edge sites, thereby inhibiting CH_4_ production.^21^ Therefore, these results suggest that the investigation of synergistic interactions between MoS_2_ and other promoter compounds deserves closer attention.

In earlier works, MoS_2_ has been combined with K,^22^ Co (ref. 23) and Ni (ref. 24) promoters leading to enhanced CO hydrogenation to higher alcohols at pressures around 100 bar. More recently, these promoters were incorporated into the hydrothermal synthesis of MoS_2_, leading to considerable changes in its catalytic activity for CO and CO_2_ hydrogenation, as the selectivity can be shifted from CH_4_ to CO under lower reaction pressures of 20 bar.^25^

In this work, K and Co promoted-MoS_2_ are compared with Mn-promoted MoS_2_ produced by a hydrothermal method. Following catalytic testing for CO_2_ hydrogenation, Mn-promoted MoS_2_ is further characterized by XRD, EXAFS, SEM, EDX, and XPS methods, seeking to investigate the main active phases that drive methanol production in the material.

## Experimental

### Catalyst synthesis

Mn-promoted MoS_2_ was produced by a hydrothermal method, in which 2.2 g of ammonium heptamolybdate tetrahydrate ((NH_4_)_6_Mo_7_O_24_·4H_2_O, Carl Roth, 99%) was dissolved in approximately 20 mL water, together with the appropriate amounts of thiourea (CH_4_N_2_S, Merck, 99%) and manganese sulfate monohydrate (MnSO_4_·H_2_O, Merck, 99%). After stirring for 30 minutes, the mixture is transferred to an autoclave and kept at 200 °C during 16 h. The precipitate was filtered, washed with water and ethanol, dried under vacuum at room temperature and finally calcined under N_2_ for 3 h.

For the comparison of pure MoS_2_ with different promoters, Mn(0.5)–MoS_2_, whose nomenclature refers to the nominal Mn/Mo molar ratio of 0.5, was produced using a CH_4_N_2_S : MnSO_4_·H_2_O : (NH_4_)_6_Mo_7_O_24_·4H_2_O molar ratio of 32 : 3.5 : 1 and a calcination temperature of 500 °C. The analogous synthesis of K(0.5)–MoS_2_, Co(0.5)–MoS_2_ and MoS_2_ is reported elsewhere.^25^ The optimized Mn(0.3)–MoS_2_ was produced using a CH_4_N_2_S : MnSO_4_·H_2_O : (NH_4_)_6_Mo_7_O_24_·4H_2_O molar ratio of 24 : 2.1 : 1 and a calcination temperature of 400 °C.

### Material characterization

X-ray powder diffraction (XRD) was performed in a PANalytical Empyrean. A Cu-LLF X-ray tube (45 kV, 40 mA, CuKα *λ*_1_ = 1.5406 A, *λ*_2_ = 1.5444 A) and a GaliPIX detector were used in Bragg–Brentano geometry. Scanning Electron Microscopy (SEM) was carried out using a FEI Quanta 250 FEG at a 5 kV voltage. Attached to the same device is an Octane Elite Super detector, which was employed for Energy Dispersive X-Ray (EDX) analysis, carried out at 20 kV.

Near-Ambient-Pressure X-ray Photoelectron Spectroscopy (NAP-XPS) was performed with a near ambient-pressure XPS system from SPECS (AlKα source, Phoibos 150 NAP detector) using a sample stage optimized for catalytic measurements.^26^ The sample was mounted on a quartz sample carrier with steel backplate and heated with a laser. Pretreatment was carried out at 0.75 mbar H_2_ and 400 °C and reaction conditions were simulated under 1 mbar H_2_ : CO_2_ = 3 : 1 at 200 °C. *Ex situ* XPS was carried out using a SPECS u-Focus system (AlKα source, Phoibos 150 WAL detector). XPS data evaluation was carried out with CasaXPS software.^27^ The peaks were fitted with Gauss–Lorentz (GL) sum functions and a Shirley background was used. All spectra were calibrated for S 2p_3/2_ = 162.0 eV.^28,29^

XAS spectra of the Mn and Mo K-edge were collected at the SuperXAS beamline at PSI. It operated in top-up mode at 2.4 GeV and a ring current of 400 mA. A silicon-coated mirror (which also reduced higher-order harmonics) was used to collimate the polychromatic X-rays from a 2.9 T superbend magnet, which were subsequently monochromatized by a Si(111) channel-cut crystal. The monochromator was rocked at a frequency of 1 Hz resulting in two spectra per second. For Mn K-edge a Si-coated and for Mo K-edge a Rh-coated toroidal mirror focused the beam. The Mn, Mo K-edge absorption spectra were collected in transmission mode using 15 cm long ionization chambers filled with 1 bar of N_2_ for Mn and 1 bar of N_2_ plus 1 bar of Ar for Mo. A metal foil was measured simultaneously for absolute energy calibration. Standard background subtraction, interpolation, and averaging were done with the Python-based software ProQEXAFS.^30^ Normalization and analysis were performed with Demeter.^31^ The samples were measured in pellet form diluted by cellulose for optimized signal. In addition to the catalysts, commercial samples of MnO (ABCR, 99%) and MnCO_3_ (ABCR, 95%) were analyzed for comparison.

### Catalytic measurements

The catalytic activity was measured on a “micro effi” system of PID Eng&Tech in a fixed bed plug flow steel reactor. For the measurements, 1 g of the pure catalyst was pretreated at 21 bar H_2_ at 400 °C. The reaction was conducted for 8 h at each temperature under 20% CO_2_, 60% H_2_ and 20% He at 21 bar, with a total flow of 5 mL_n_ min^−1^. The product gas was analyzed by an Inficon Micro GC 3000 using a Plot Q column.

## Results and discussion

**Fig. 1:**
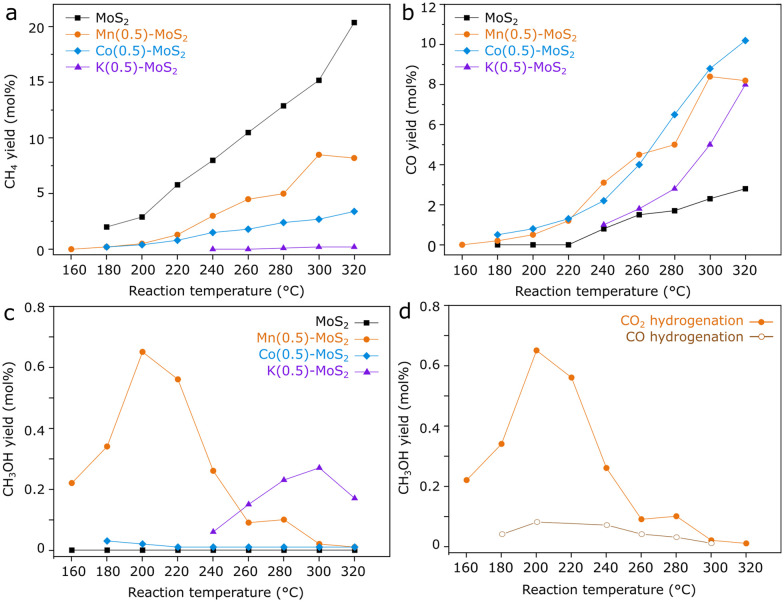
a) CH_4_, b) CO, c) CH_3_OH yield obtained frm catalytic reaction with 1 g MoS_2_, Mn(0.5)-, Co(0.5)-, and K(0.5)-promoted MoS_2_ under 1 mL min^−1^ CO_2_ + 3 mL min^−1^ H_2_ + 1 mL min^−1^ He at 21 bar and d) CH_3_OH yield under CO_2_ hydrogenation conditions, compared with an analogous experiment using CO as carbon source.

Seeking to identify potentially promising promoters for MoS_2_ catalysts in CO_2_ hydrogenation, samples of M-promoted MoS_2_ (M = K, Co or Mn) have been produced by analogous hydrothermal approaches. Subsequently, their catalytic activity for CO_2_ hydrogenation was compared with pure MoS_2_ obtained by the same method, as shown in Fig. 1a–c.

As expected, pure MoS_2_ shows high selectivity for CH_4_ between 180 and 320 °C, although the activity for CO production through the reverse water-gas-shift reaction becomes prominent above 220 °C. In contrast, the incorporation of K, Co or Mn precursors clearly lowers CH_4_ and increases CO yields with respect to the pure material over the entire temperature range. Furthermore, Co–MoS_2_ exhibits negligible methanol yield, while in K–MoS_2_ some methanol production is observed around 280 °C.

Mn-promoted MoS_2_, however, shows a distinct performance, with the highest methanol yield occurring at much lower temperatures around 200 °C. While pure MoS_2_ shows a negligible methanol yield at 180 °C, Mn(0.5)–MoS_2_ produces methanol with a selectivity of 45% at a CO_2_ conversion of 0.8% under the same conditions. Furthermore, Fig. 1d shows that methanol production is mostly suppressed when CO is alternatively used as the feed gas, demonstrating that the catalyst cannot produce methanol from CO hydrogenation under these conditions. This observation suggests that a reaction mechanism involving CO as an intermediate can be ruled out.

Therefore, it is likely that methanol is produced *via* direct CO_2_ hydrogenation *via* formate pathway in the presence of the Mn-promoted MoS_2_ catalyst. Although this finding contrasts with the previously demonstrated CO-based pathway observed for pure MoS_2_ nanosheets,^20^ the coexistence of formate and CO intermediates has been already suggested for the MoS_2_/ZnS catalyst.^21^ The divergence in these results support the idea that CO_2_ hydrogenation to methanol in MoS_2_-based catalysts may undergo distinct pathways due to structural changes and the addition of promoters to MoS_2_, similarly as observed for copper-based catalysts, for example.^11–14^

To obtain preliminary insights on the nature of the Mn promoter, characterization of Mn(0.5)-MoS_2_ was conducted by XRD and *in situ* NAP-XPS. In Fig. S1,† the XRD pattern of Mn(0.5)–MoS_2_ shows that the catalyst crystalline structure consists mostly of MoS_2_,^32^ MnO,^33^ and MnO_2_.^34^ Differently from previously reported K- and Co-promoted MoS_2_,^25^ no other sulfides and sulfates are formed in abundance, as MnS (ref. 35) appears with very low crystallinity in the XRD pattern.

**Fig. 2:**
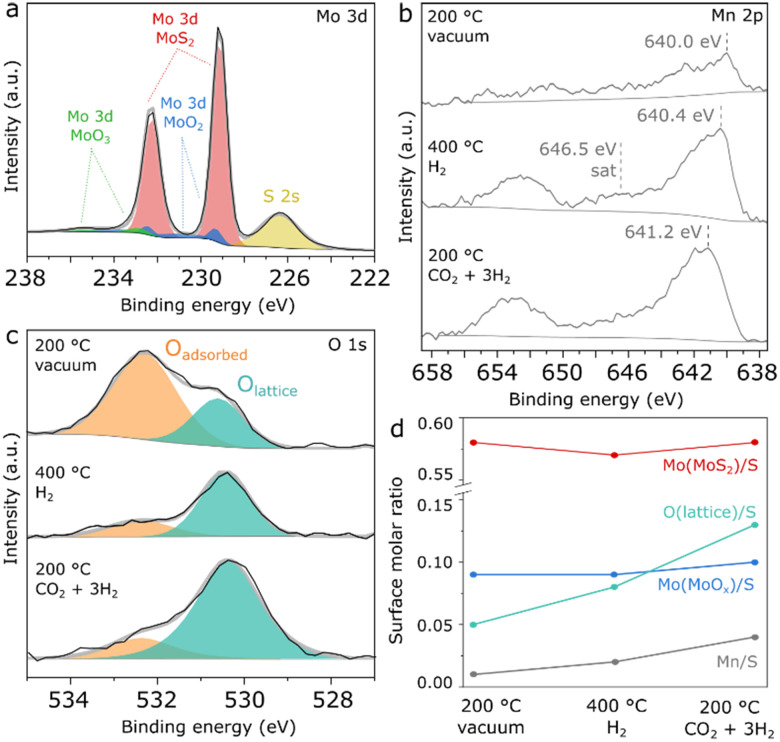
NAP-XPS spectra of Mn(0.5)–MoS_2_ showing the regions a) Mo 3d under vacuum at 200 °C, b) Mn 2p and c) O 1s under vacuum at 200 °C, H_2_ at 400 °C and CO_2_ + 3H_2_ at 200 °C and d) summarized quantification of surface molar ratios with respect to S.

In order to obtain more detailed insights into the surface composition of Mn(0.5)–MoS_2_ under reaction-relevant conditions, NAP-XPS analysis was carried out firstly under vacuum at 200 °C, followed by H_2_ flow at 400 °C and CO_2_ + 3H_2_ flow at 200 °C. Fig. 2a shows the region comprising Mo 3d and S 2s spectra. A single contribution is observed for sulfur at 226.4 eV, as typically reported for S^2−^ in MoS_2_.^28,29^ The Mo 3d region, however, shows a more complex profile with three distinct species, which were fitted in accordance with the characteristic doublet separation of 3.14 eV. Coherently with the observation from XRD, the main Mo surface species consist of Mo^4+^ associated with MoS_2_, evidenced by the 3d_5/2_ peak at 229.1 eV.^28,29^ Within a similar energy range, Mo^4+^ from MoO_2_ is described by two neighboring doublets with different widths, in order to account for the typical asymmetry of MoO_2_ peaks arising from the distinctive narrow band metallic character of this compound.^36^ Accordingly, this species is fitted with neighboring 3d_5/2_ peaks at 229.3 eV and 231.0 eV, and respective 3d_3/2_ counterparts following area, width and position constraints reported in a previous study. Furthermore, a minor contribution from another doublet is verified, with a 3d_5/2_ peak at 232.9 eV as a possible result of surface Mo^6+^ oxides.^36,37^ Correspondingly, MoS_2_ produced by an analogous hydrothermal approach without the Mn promoter also presents similar evidences for surface Mo oxides, as seen in Fig. S2.† Since this pure MoS_2_ sample has shown negligible methanol selectivity, this finding suggests that MoO_*x*_ should not be responsible for the promoting effect.

As shown in Fig. S3,† the chemical environment of surface Mo and S experiences insignificant changes upon H_2_ pretreatment and reaction conditions. In Fig. 2b, the Mn 2p region initially shows a doublet with very low intensity, which suggests a low surface concentration of the Mn promoter. However, under H_2_ treatment at 400 °C, the Mn 2p doublet becomes more prominent, exhibiting a 2p_3/2_ maximum at 640.4 eV and a satellite feature around 646.5 eV, as possible indicators of Mn^2+^ in MnO.^38^ Subsequently, under CO_2_ + 3H_2_ flow at 200 °C the Mn 2p doublet is shifted towards higher binding energy by almost 1 eV, suggesting the formation of higher oxidation states such as Mn^3+^ and Mn^4+^ at the surface under reaction conditions. However, precise determination and quantification of these phases is challenging in a spectrum with such low intensity due to the complex and asymmetric character of the components associated with manganese oxides.^38^

In Fig. 2c, the O 1s spectrum shows surface oxygen described by two species: the component at approximately 530.6 eV can be associated with lattice oxygen from Mo or Mn oxides, while the one at 532.3 eV typically refers to surface hydroxyl or organic species.^39^ Although initially the lattice oxygen component is smaller, it becomes much more prominent than the adsorbed species under pretreatment and reaction conditions.

As a summary of the NAP-XPS analysis, Fig. 2d shows a quantification based on combined survey spectra and high-resolution Mo 3d and O 1s regions. While the components related to MoS_2_ and MoO_*x*_ show negligible changes, the Mn/S surface molar ratio increases concurrently with the O_lattice_/S ratio during H_2_ pretreatment and reaction conditions, suggesting the formation of surface Mn oxides at the surface under reaction-relevant conditions. Interestingly, Mn^2+^ appears to coexist with higher Mn oxidation states despite the reducing environment created by H_2_ and CO_2_, as a possible consequence of oxygen transferred from bulk to surface or due to the dissociation of CO_2_ on MnO. Although precise identification of this surface oxidation mechanism is challenging, NAP-XPS analysis suggests that Mn oxides are the key Mn-containing phase under reaction conditions.

Given the promising catalytic activity of Mn-promoted MoS_2_, the hydrothermal synthesis was further optimized in terms of calcination temperature, content of thiourea and content of Mn precursor. As shown in Fig. S4,† the methanol yield is improved by lowering calcination temperature from 500 °C to 400 °C, the MnSO_4_·H_2_O : (NH_4_)_6_Mo_7_O_24_·4H_2_O molar ratio from 3.5 : 1 to 2.1 : 1 and CH_4_N_2_S : (NH_4_)_6_Mo_7_O_24_·4H_2_O from 32 : 1 to 24 : 1.

**Fig. 3:**
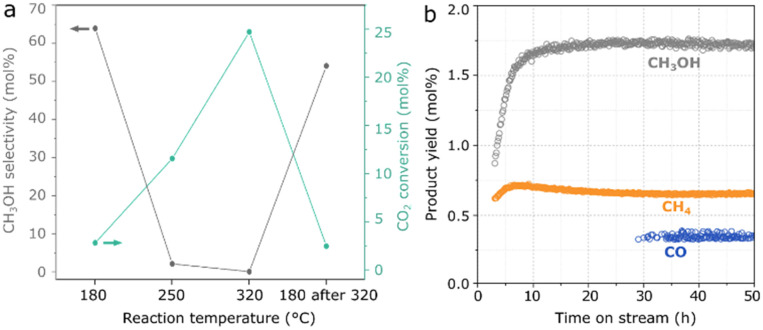
Methanol selectivity and total CO_2_ conversion as a result of a) 8 h catalytic reaction at 180 °C, 250 °C, 320 °C and 180 °C after 320 °C with 1 g Mn(0.3)–MoS_2_ under 1 mL min^−1^ CO_2_ + 3 mL min^−1^ H_2_ + 1 mL min^−1^ He at 21 bar and b) CH_3_OH, CH_4_ and CO yields under the same conditions during a 50-hour reaction.

In Fig. 3a, as a result of the optimized hydrothermal method, Mn(0.3)–MoS_2_ shows a methanol selectivity of 64% with an improved CO_2_ conversion of 2.8% at 180 °C. At higher temperatures, the formation of CO and CH_4_ becomes dominant, although further cooling to 180 °C does not lead to an expressive decrease in methanol yield and selectivity. Furthermore, the material also exhibits stable catalytic activity at 180 °C during a 50-hour experiment, as shown in Fig. 3b.

**Fig. 4:**
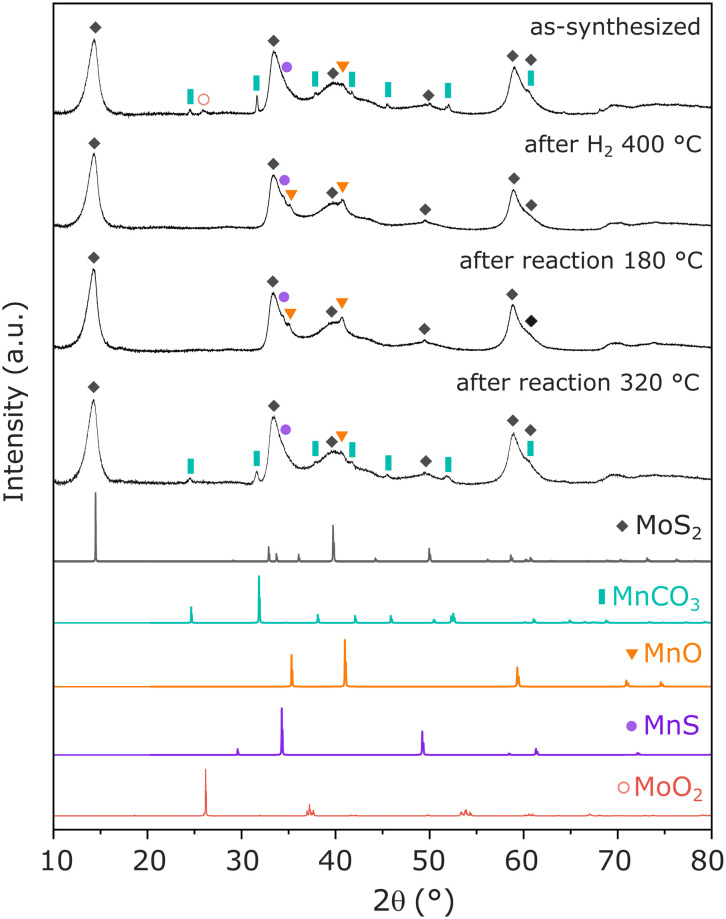
XRD pattern of Mn(0.3)–MoS_2_ following synthesis, H_2_ pretreatment at 400 °C and catalytic reaction at 180 °C and 320 °C, in comparison with reference data for MoS_2_ (COD-ID 1010993), MnCO_3_ (COD-ID 1011228), MnO (COD-ID 1514099), MnS (COD-ID 1011351) and MoO_2_ (COD-ID 9009090).

To identify the main Mn-containing phases in the optimized catalyst, XRD and EXAFS have been performed in Mn(0.3)–MoS_2_ catalyst, following exposure to relevant reaction conditions. In Fig. 4, as-synthesized Mn(0.3)–MoS_2_ presents a variety of phases. As expected, the main contribution consists of MoS_2_ (ref. 32) with low crystallinity, which is a typical outcome from the hydrothermal method. The promoter Mn is mostly present in the form of MnCO_3_ (ref. 40) as a result of the reaction between the Mn precursor and thiourea during the hydrothermal synthesis. Additionally, the diffractogram suggests that minor contributions from MoO_2_ (ref. 32) and nearly amorphous MnO (ref. 33) and MnS (ref. 35) are also present in the material.

The pretreatment under H_2_ at 400 °C significantly alters the catalyst structure: although the patterns related to MoS_2_ and MnS do not experience significant changes, MnCO_3_ is absent in the pretreated material while a noticeable profile related to MnO (ref. 33) arises. This observation indicates that the pretreatment converts MnCO_3_ into MnO, in an analogous manner as previously reported under H_2_ at similar temperatures.^41,42^ Furthermore, the feature related to MoO_2_ disappears, possibly indicating amorphization under reducing conditions. All these features are maintained after the material undergoes catalytic reaction at 180 °C, demonstrated to be the optimal condition for CO_2_ hydrogenation to methanol.

Moreover, after reaction at 320 °C, the material exhibits once again the pattern from MnCO_3_, indicating that the higher temperature favors the carbonation reaction of MnO in the presence of the CO_2_ + 3H_2_ mixture at 21 bar. This phase transformation may be closely associated with the slight catalyst deactivation after reaction at 320 °C, already shown in Fig. 3a.

Given the observation of MnO following catalytic reaction at the ideal conditions for methanol production, this phase can be pointed out as a likely key feature behind the promoting effect of Mn. Nevertheless, due to the possible effect of amorphous Mn-containing species that could remain undetected by XRD, such as other oxides, sulfides or Mn intercalated within MoS_2_ layers, EXAFS analysis was carried out in order to elucidate the coordination environment of Mn atoms in the catalyst.

**Fig. 5:**
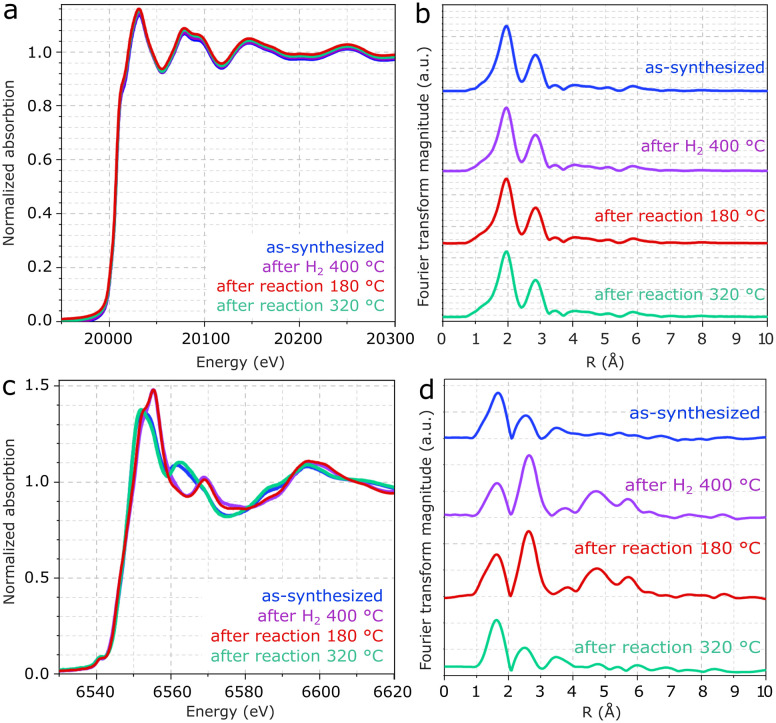
a and b) XANES spectra and the associated EXAFS analysis of the Mo and c and d) Mn K-edges from Mn(0.3)–MoS_2_ after synthesis, H_2_ pretreatment at 400 °C and catalytic reaction at 180 °C and 320 °C.

In Fig. 5a, XANES spectra of the Mo edge show negligible differences between the samples. Fig. 5b and S5† show that the Fourier transform of the Mo K-edge exhibits mainly two features related to Mo–S and Mo–Mo, coherently with MoS_2_.^43^ As suggested in the EXAFS fitting in Fig. S5 and Table S1,† the higher prominence of the Mo-S coordination with respect to Mo–Mo may be associated with the low crystallinity of MoS_2_, in line with the XRD patterns and previous EXAFS reports.^44,45^ Despite the differences observed for Mn, the Mo K-edges remain unchanged regardless of exposure to pretreatment and reaction conditions. Therefore, the result confirms the high stability of MoS_2_ following reaction conditions and rules out major contributions from Mo oxides to the catalyst composition.

As shown in Fig. 5c, XANES spectra from the Mn K-edge region of Mn(0.3)MoS_2_ give evidence for considerable changes between as-synthesized and spent catalysts. In Fig. 5d, the corresponding Fourier transforms show 2 main features related to Mn–O and Mn–Mn coordination shells.^46,48^ Consistently with the presence of MnO observed by XRD after treatment with H_2_ at 400 °C and reaction at 180 °C, the EXAFS spectrum clearly shows the characteristic profile associated with MnO (ref. 47 and 48) in these two samples, as confirmed by the fitting presented in Fig. S6 and Table S2.†^43,45^

**Fig. 6:**
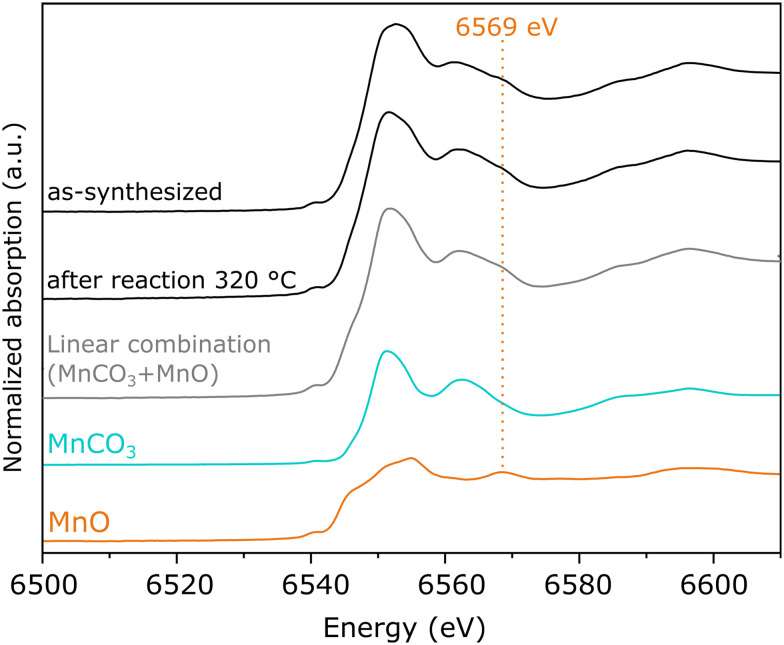
XANES spectra of the Mn K-edge from Mn(0.3)–MoS_2_ after synthesis and catalytic reaction at 320 ° C, compared to MnO and MnCO_3_ references samples and their linear combination considering equal proportions of each phase.

On the other hand, as-synthesized Mn(0.3)MoS_2_ exhibits a similar XANES spectrum from the one observed after 320 °C reaction, as shown in Fig. 5c. Despite some similarity with the typical XANES profile observed in MnCO_3_,^49^ the respective EXAFS data shows considerable differences when compared to a MnCO_3_ reference in Fig. S7,† which raises the possibility of a more complex composition. In fact, Fig. 6 shows that these XANES spectra may be described as a linear combination of MnCO_3_ and MnO. A clear indication of this similarity is the distinctive feature of MnO around 6569 eV, which makes the XANES spectra easily distinguishable from those of MnS and other Mn oxides.^50^ In view of this finding, as-synthesized Mn(0.3)MoS_2_ is considered to present the Mn promoter in the form of polycrystalline MnCO_3_ in combination with low-crystallinity MnO, similarly as observed for the material exposed to reaction at 320 °C, since both samples show a weak contribution of the oxide in the XRD patterns in Fig. 4.

Material characterization by XRD and XAS strongly suggests that MoS_2_ and MnO are the key components related to CO_2_ hydrogenation to methanol in this catalyst. In order to unveil how these phases are present at the catalyst surface, further characterization was performed by SEM and XPS.

**Fig. 7:**
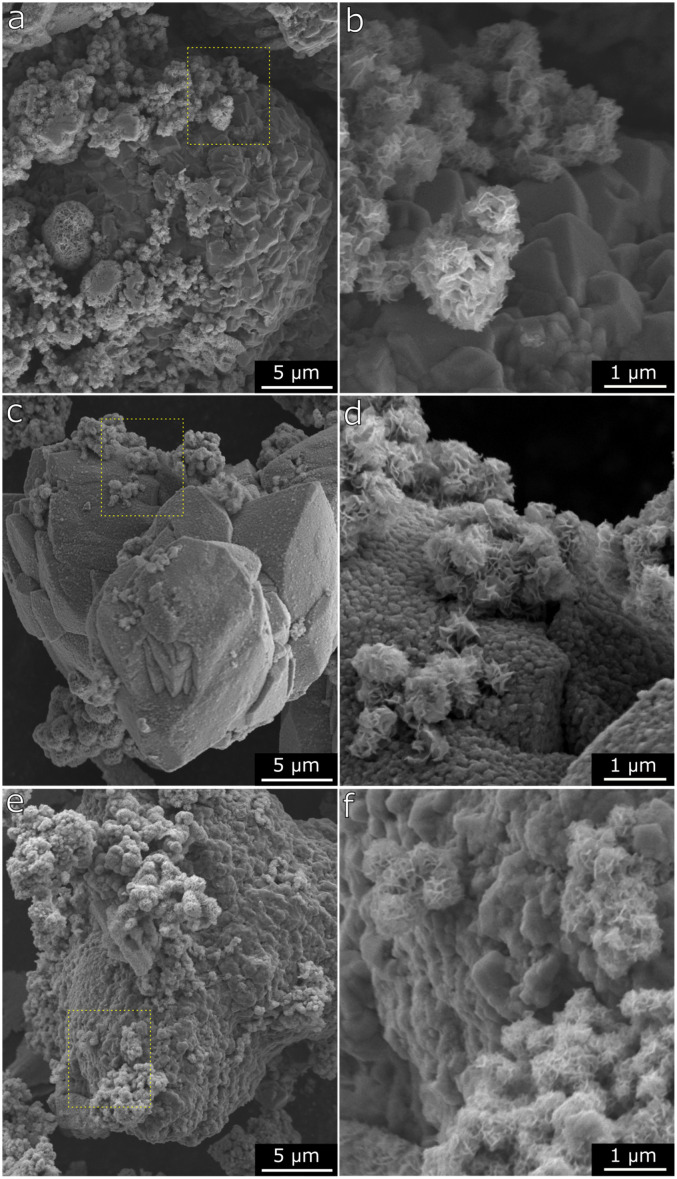
a and b) SEM micrographs of Mn(0.3)–MoS_2_ after synthesis, c and d) after H_2_ pretreatment at 400 °C and reaction at 180 °C, e and f) after H_2_ pretreatment at 400 °C and reaction at 320 °C.

Fig. 7a and S8† show that the Mn(0.3)MoS_2_ surface is mostly composed of thin MoS_2_ sheets arranged in a nanoflower morphology, highly similar to MoS_2_ produced by an analogous hydrothermal synthesis. This aspect is consistent with the broad XRD pattern observed for MoS_2_, which suggests small crystallites and sparse stacking of MoS_2_ layers. Moreover, these features coexist with another evident morphology consisting of the micrometer-sized particles shown in more detail in Fig. 6b. After H_2_ pretreatment and subsequent catalytic reaction at 180 °C, the nanoflower structure associated with MoS_2_ remains unchanged, as shown in Fig. 6c and d. On the other hand, the larger particles are significantly altered, now featuring a rougher surface with abundant pores in the nanometer range. This finding is consistent with the conversion of MnCO_3_ into MnO, which releases CO and CO_2_, thus forming the characteristic porous surface. Such morphology change has also been already reported following exposure of MnCO_3_ to reducing conditions under similar temperatures.^41,42^ As demonstrated by XRD and EXAFS analysis of Mn(0.3)MoS_2_, further increasing reaction temperature to 320 °C induces the partial carbonation of MnO into MnCO_3_. This effect can also be correlated with the SEM data in Fig. 6e and f, where the characteristic porous surface of MnO is again less prominent.

**Fig. 8:**
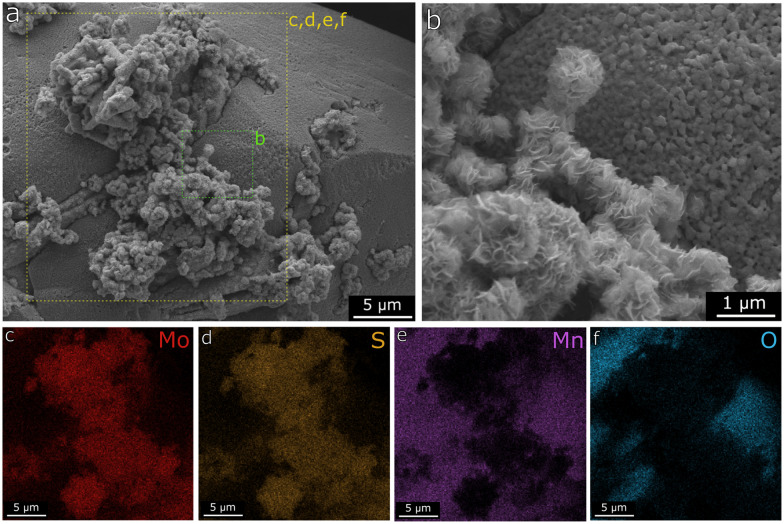
a and b) SEM micrograph with the respective EDX element mapping for c) Mo, d) S, e) Mn and f) O atoms for Mn(0.3)–MoS_2_, after H_2_ pretreatment at 400 °C.

Further evidence for the MoS_2_/MnO system after H_2_ pretreatment is shown in Fig. 8, in which the EDX mapping confirms the elemental composition of the nanosheets as mostly Mo and S, while the larger particles are rich in Mn and O. A summary of the EDX spectra contained in the evaluated region is displayed in Fig. S9.†

**Fig. 9:**
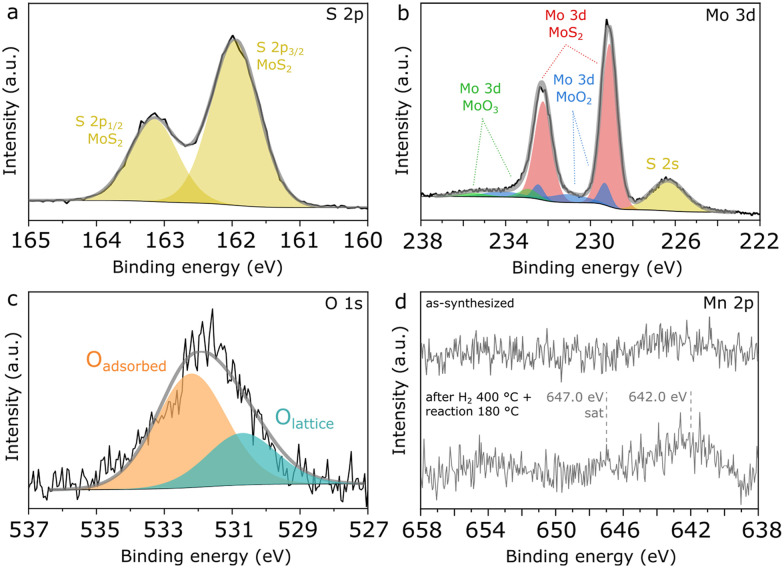
High-resolution XPS spectra of Mn(0.3)–MoS_2_ showing a) S 2p, b) Mo 3d, c) O 1s and d) Mn 2p regions.

Finally, further insights into the surface composition of Mn(0.3)–MoS_2_ are provided by XPS analysis of fresh and used catalysts. In Fig. 9a, the S 2p spectrum shows a unique doublet with the characteristic splitting of 1.2 eV and S 2p_3/2_ located at 162.0 eV, as typically reported for sulfides such as MoS_2_. Similarly as observed in the NAP-XPS analysis of Mn(0.5)–MoS_2_, Fig. 9b indicates that MoS_2_ (Mo 3d_5/2_ at 229.1 eV)^28,29^ coexists with surface MoO_2_ (Mo 3d_5/2_ at 229.3 eV and 231.0 eV) as well as MoO_3_ (Mo 3d_5/2_ at 232.9 eV),^36,37^ although MoS_2_ brings again the most prominent contribution.

Accordingly, Fig. 9c shows that the O 1s spectrum indicates two distinct contributions: the minor peak at 532.3 eV can be ascribed to adsorbed oxygen from water or surface hydroxyl species, while the most prominent is located at 530.6 eV, associated with Mo and Mn oxides.^39^ The low intensity of the peak related to lattice oxygen can be correlated with the minor contributions from oxides in the Mo 3d and Mn 2p spectra, thus confirming that metal oxides consist only of a minor contribution to the total surface composition, with MoS_2_ being the dominant species.

In order to verify possible modifications at the surface of Mn(0.3)MoS_2_, XPS analysis was also conducted after H_2_ pretreatment at 400 °C and reaction at 180 °C. As shown in Fig. S10,† these conditions do not promote expressive changes in S 2p, Mo 3d and O 1s regions, suggesting that the surface remains rich in stable MoS_2_, in line with the strong similarities in the nanosheet morphology observed by SEM both before and after reaction. On the other hand, Fig. 9d shows that a significant change is observed in the Mn 2p region, as the doublet is only visible in the spent catalyst. This effect may be associated with the phase transition from MnCO_3_ to MnO experienced by the catalyst upon H_2_ pretreatment, as the higher surface area of the porous MnO could enhance the photoelectron signal related to the Mn species. Even after pretreatment and reaction, the Mn/Mo surface atomic ratio is still approximately 0.1, much lower than the nominal value of 0.3, as a possible outcome of the extensive covering of Mn phases by MoS_2_ sheets demonstrated by the SEM data.

Furthermore, in the spent catalyst the Mn 2p spectrum is consistent with concurrent MnO and MnO_*x*_ with higher oxidation states, given the combination of a faint satellite feature related to MnO near 647.0 eV and the 2p_3/2_ peak located around 242.0 eV.^38^ Since XRD and EXAFS characterization give evidence of MnO as the only Mn oxide phase in the material, these oxidized MnO_*x*_ species are understood to be limited to the catalyst surface.

In previous research, S-vacancies in MoS_2_ have been strongly associated with its catalytic activity, and their presence at the surface can be usually verified by XPS. According to quantification based on Mo 3d and S 2s regions shown in Fig. 9b, an approximate S/Mo ratio of 1.7 is calculated considering only the Mo component associated with MoS_2_. This low value may be associated with abundant sulfur vacancies formed during calcination under N_2_, as it does not change significantly after H_2_ pretreatment and reaction.

Moreover, given that abundant basal plane sulfur vacancies have been strongly suggested as active sites for CO_2_ hydrogenation to methanol,^20^ an oxygen chemisorption experiment coupled with *in situ* DRIFTS has been performed in an attempt to distinguish between edge- and basal plane sulfur vacancies. However, as shown in Fig. S11,† the results indicate no visible changes in the vibrational spectrum before and after O_2_ flow. Although this suggests a limited concentration of basal plane sulfur vacancies in the catalyst with respect to previous reports, the presence of other surface metal oxides such as MoO_*x*_ and MnO_*x*_ introduces overlapping vibrational bands that may hinder the detection of the typical features related to oxygen chemisorption on sulfur vacancies.

In summary, characterization of Mn(0.3)–MoS_2_ upon hydrothermal synthesis indicates that the Mn promoter is initially present as a combination of MnCO_3_ and low-crystallinity MnO. Subsequently, H_2_ pretreatment fully converts the carbonate into MnO, although higher oxidation states for Mn may be present at the surface. This MoS_2_/MnO_*x*_ character is maintained after reaction at 180 °C. Even though increasing reaction temperature to 320 °C leads to partial carbonation of MnO, only a mild catalyst deactivation is observed.

Despite the correlation of MoS_2_/MnO_*x*_ with catalytic activity, it is challenging to evaluate if MnCO_3_ plays any role in catalytic activity, as here this phase always coexists with MnO, as demonstrated by XANES analysis. In light of these findings, CO_2_ hydrogenation to methanol may be associated with the synergy between MoS_2_ and MnO_*x*_, while pure MoS_2_ obtained by an analogous synthesis approach shows negligible selectivity for methanol.

In view of recent findings, MoS_2_ growth in the vicinity of MnO_*x*_ might explain the improved methanol selectivity, since edge-blocking effects could inhibit the production of CH_4_ in MoS_2_ edges.^21^ Therefore, further studies with simpler MoS_2_/MnO_*x*_ systems and detailed characterization of S-vacancies will be important for understanding the interplay between MoS_2_ and MnO_*x*_ during CO_2_ hydrogenation to methanol.

In comparison with other MoS_2_-based materials presented in Table S3† such as few-layer MoS_2_,^20^ MoS_2_/ZnS (ref. 21) and Cu/MoS_2_@SiO_2_,^51^ the Mn(0.3)–MoS_2_ catalyst has a moderate methanol selectivity at lower or comparable CO_2_ conversion levels. Accordingly, as shown in the SEM results, MoS_2_ is not well dispersed with the Mn promoter phase, which would explain the notable production of the CH_4_ byproduct related to pure MoS_2_. Therefore, this suggests MoS_2_/MnO_*x*_ catalysts could be further improved by employing synthesis methods that enhance the dispersion of these phases and their surface area.

## Conclusions

In summary, this work demonstrates that Mn-promoted MoS_2_ presents promising properties as a catalyst for CO_2_ hydrogenation to methanol. Even though its catalytic activity is lower than in some other MoS_2_-based catalysts, a sharp increase in methanol selectivity is observed in comparison with pure MoS_2_ obtained by an analogous hydrothermal synthesis method. This improvement suggests a promoting effect of Mn, which may be closely associated with the presence of Mn oxides, according to material characterization. More specifically, the optimized catalyst contains MnO as the main Mn phase, although surface characterization indicates that Mn^2+^ coexists with higher oxidation states under reaction-relevant conditions. This finding suggests that the limited selectivity to methanol could be further improved in catalysts with abundant MoS_2_/MnO_*x*_ interfaces.

Furthermore, given the importance of basal plane S-vacancies in MoS_2_ catalysts for CO_2_ hydrogenation to methanol,^20^ it can be speculated that MoS_2_/MnO_*x*_ interfaces facilitate this reaction in Mn-promoted MoS_2_, possibly due the blockage of MoS_2_ edge sites, as previously observed for a MoS_2_/ZnS system.^21^ Therefore, further characterization focused on S-vacancies is necessary for a deeper understanding of such edge-blocking mechanism between MoS_2_ and metal-oxides. Moreover, the evidence for direct CO_2_ hydrogenation without a CO intermediate in Mn-promoted MoS_2_, differently as in pure MoS_2_ nanosheets,^20^ emphasizes the importance of investigating reaction mechanisms with more detail in future work.

## Author contributions

Conceptualization: G. P., K. F. and G. A. S. A. Methodology: G. P. and K. F. Formal analysis: G. A. S. A., G. P. and S. P. Investigation: G. A. S. A., G. P., S. P., T. W., C. R. and R. R. Writing – original draft preparation: G. A. S. A. and G. P. Writing – review & editing: G. A. S. A. and K. F. Supervision: K. F. Funding acquisition: K. F.

## Conflicts of interest

There are no conflicts to declare.

## Supplementary Material

CY-014-D3CY01711G-s001
