# Supplementary material for: Mn-promoted MoS2 catalysts for CO2 hydrogenation: enhanced methanol selectivity due to MoS2/MnOx interfaces
Source: Catal Sci Technol. 2024 Feb 2;14(5):1138–47. doi: 10.1039/d3cy01711g (PMC10913851; doi:10.1039/d3cy01711g)
Supplement: CY-014-D3CY01711G-s001 [file CY-014-D3CY01711G-s001.pdf]

Electronic Supplementary Material (ESI) for Catalysis Science & Technology.
This journal is © The Royal Society of Chemistry 2024

Supplementary Information

Mn-promoted MoS_2 catalysts for CO_2 hydrogenation: enhanced methanol selectivity due to MoS_2/MnO_x interfaces

Gustavo A. S. Alves[1*], Gernot Pacholik[1*], Stephan Pollitt[2], Tobias Wagner[1], Raffael Rameshan[3], Christoph Rameshan[3], Karin Föttinger[1+]

* These authors have contributed equally to this work
+ corresponding author
1 Institute of Materials Chemistry, TU Wien, Getreidemarkt 9/BC/01, 1060 Vienna, Austria
2 Paul Scherrer Institut (PSI), Forschungsstrasse 111, 5232 Villigen, Switzerland
3 Chair of Physical Chemistry, Montanuniversität Leoben, Franz-Josef-Straße 18, 8700 Leoben, Austria

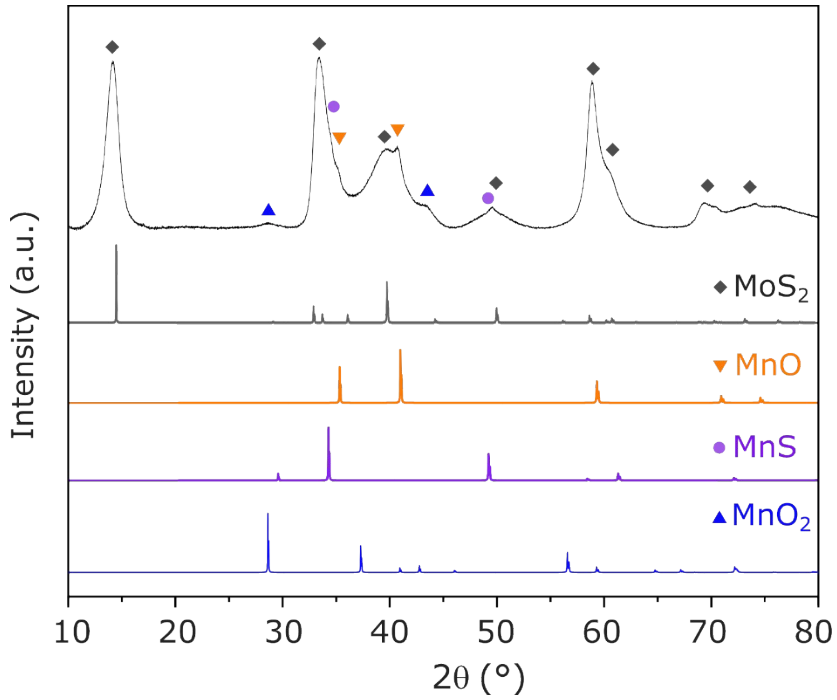

Figure S1 - XRD pattern of as-synthesized Mn(0.5)-MoS_2, compared with the references MoS_2 (COD-ID 1010993), $MnCO_3$ (COD-ID 1011228), MnO (COD-ID 1514099) and MnS (COD-ID 1011351)

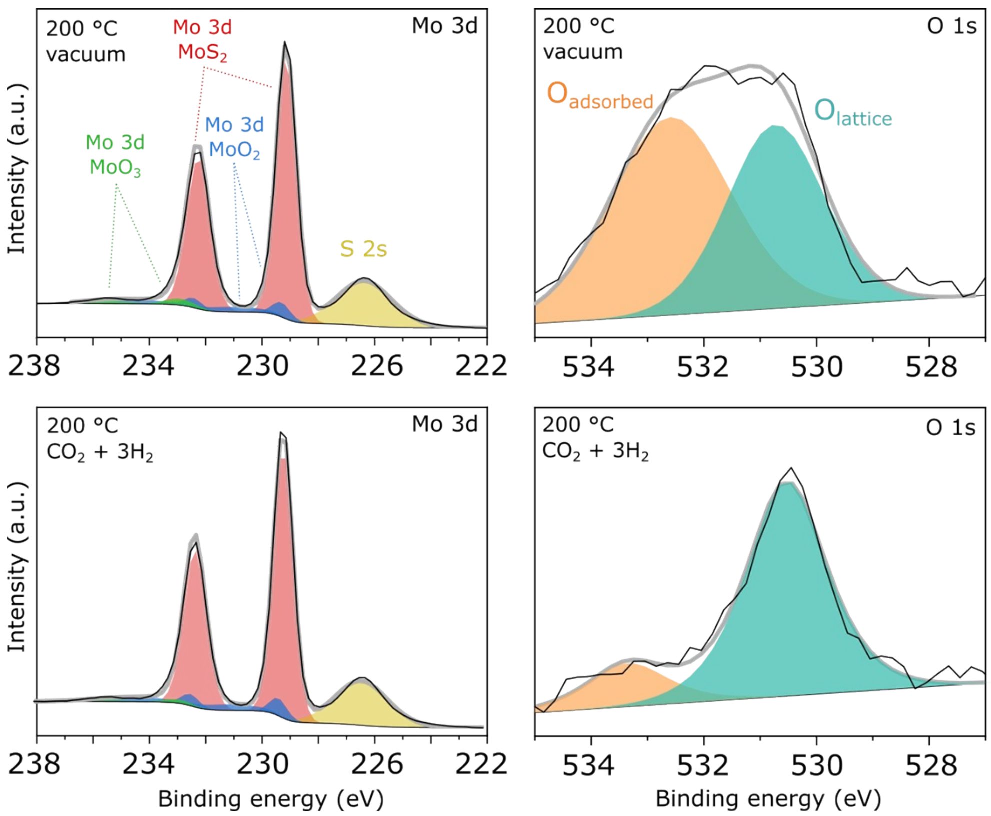

Figure S2 - NAP-XPS spectra of pure MoS_2 produced by hydrothermal synthesis, showing the regions Mo 3d and O 1s under vacuum at 200 °C, H_2 at 400 °C and CO_2+3H_2 at 200 °C

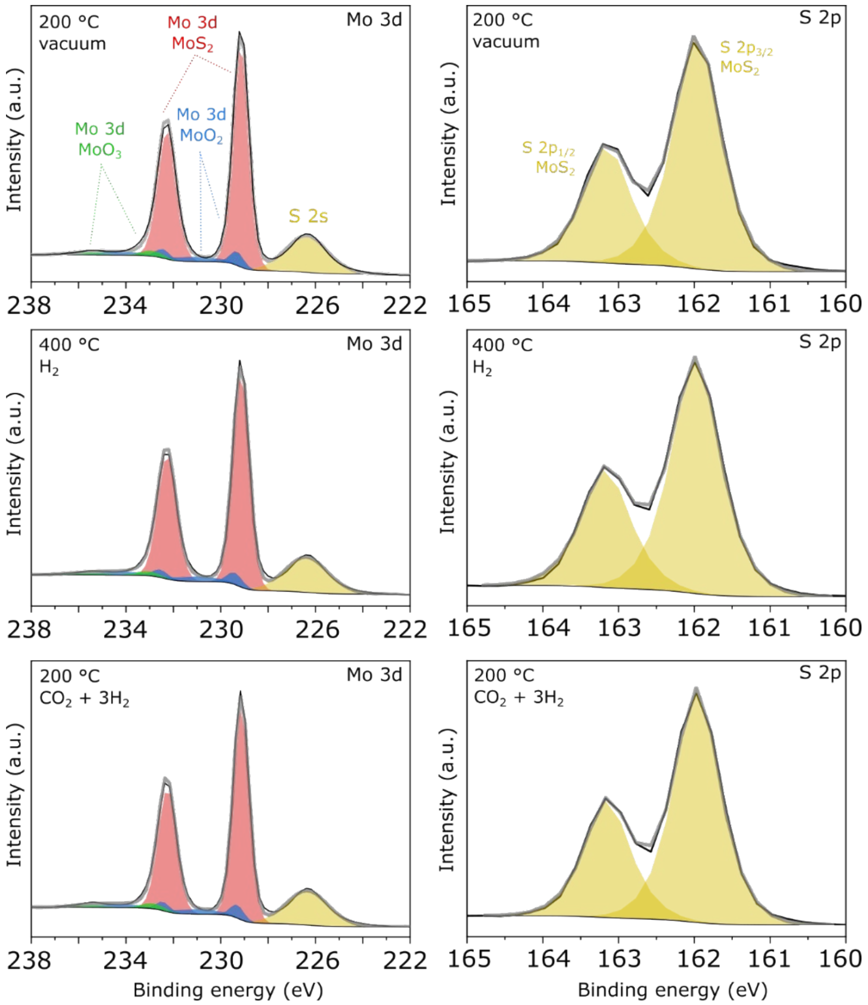

Figure S3 - NAP-XPS spectra of Mn(0.5)MoS_2 showing the regions Mo 3d and S 2p under vacuum at 200 °C, H_2 at 400 °C and CO_2+$3H_2$ at 200 °C

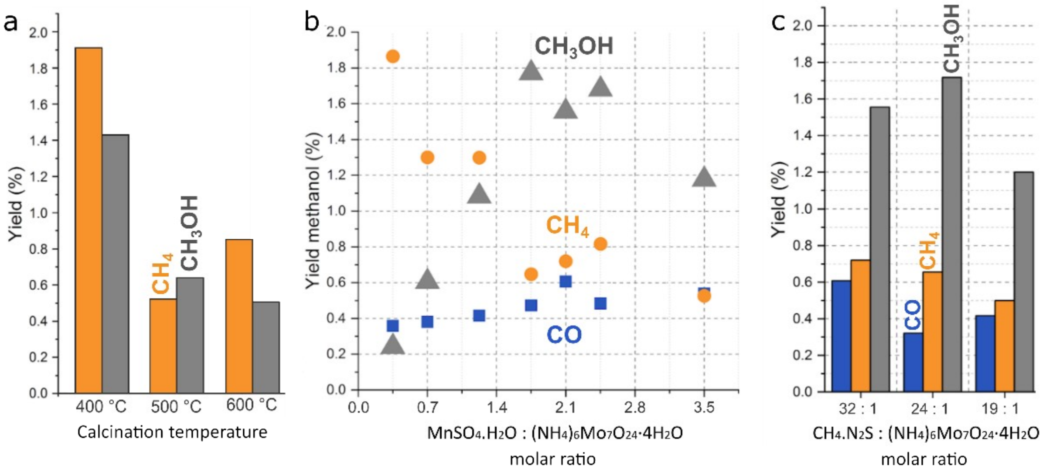

Figure S4 - Catalyst optimization, showing the product yields at 180 °C for the material synthesized using a) CH_4N_2S : $MnSO_4.H_2O$: $(NH_4)_6Mo_7O_{24}{\cdot}4H_2O$ molar ratio of 32 : 3.5 : 1, while varying the calcination temperature (CO yields were not recorded), b) CH_4N_2S : $(NH_4)_6Mo_7O_{24}{\cdot}4H_2O$ molar ratio of 4:1 with N_2 calcination at 400 °C, while varying the $MnSO_4.H_2O$: $(NH_4)_6Mo_7O_{24}{\cdot}4H_2O$ molar ratio and c) $MnSO_4.H_2O$: $(NH_4)_6Mo_7O_{24}{\cdot}4H_2O$ molar ratio of 2.1 with N_2 calcination at 400 °C, while varying the CH_4N_2S : $(NH_4)_6Mo_7O_{24}{\cdot}4H_2O$ molar ratio

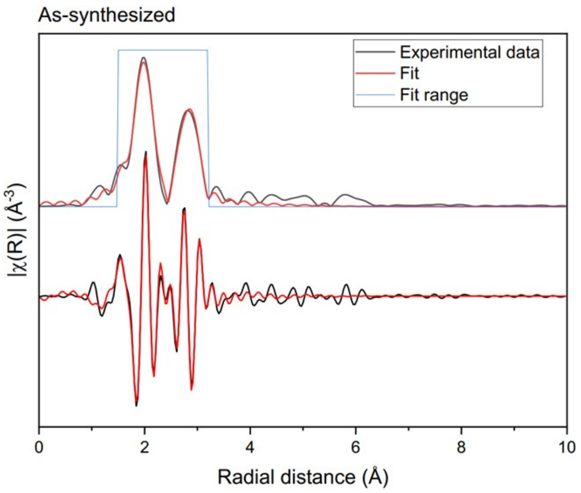

Figure S5 – EXAFS data (Mo K-edge) and fit of as-synthesized Mn(0.3)MoS_2 and the Mo foil. Fitting was performed with a K-range from 3.00 to 14 Å $^{-1}$. The amplitude reduction factor was determined by fitting of a metal foil with BCC structure[2]. The first two coordination shells were used with fixed coordination numbers (Mo-Mo1: 8, Mo-Mo2: 6). S02 was found to be 1. The experimental data was fitted in R-space in the coordination distance from 1.5 to 3.2 Å. The model was derived from a MoS_2[3] crystal structure. Two paths (Mo-S and Mo-Mo) were used for the fit.

Mo-S			Mo-Mo				
R (Å)	N	σ^2 (Å^2)	R (Å)	N	σ^2 (Å^2)	E_0 (eV)	R-factor
2.41 ± 0.02	4.8	0.003	3.17 ± 0.01	4.3	0.004	1.4 ± 1.4	0.013

Table S1 – EXAFS (Mo K-edge) fitting results of as-synthesized Mn(0.3)MoS_2

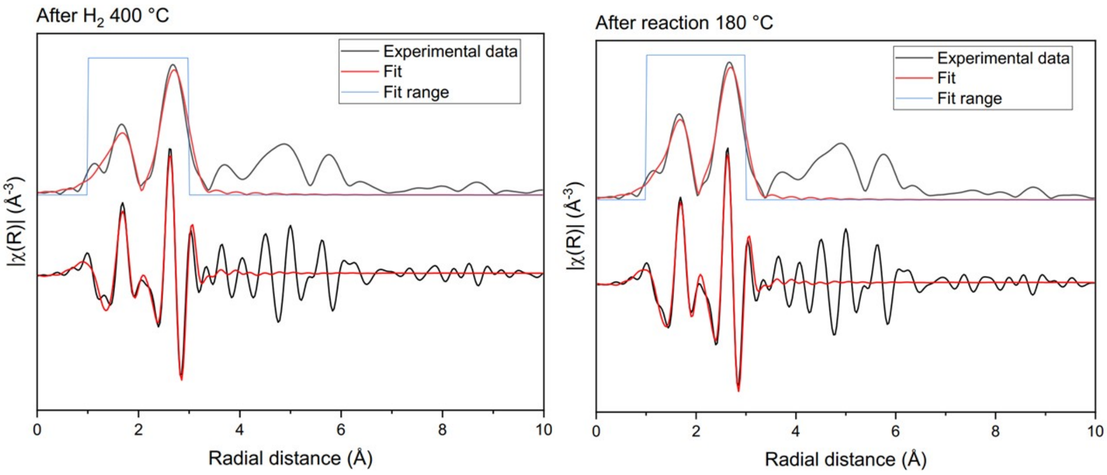

Figure S6 – EXAFS data (Mn K-edge) and fit of Mn(0.3)MoS_2 after H_2 pretreatment at 400 °C and after reaction at 180 °C. Fitting was performed with a K-range from 2.5 to 10.9 Å $^{-1}$ in the R-range of 1.00 to 3.00 Å. The reference foil was used for energy calibration but due to its excessive thickness, the amplitude reduction factor could not be obtained. The fitting of the samples was performed with the crystal structure of MnO[1] and two paths (Mn-O, Mn-Mn) with fixed coordination numbers according to the crystal structure (Mn-O: 6, Mn-Mn: 12). After fitting the sample “After H_2 400° C” the amplitude reduction factor was fixed additionally to 0.72 for the fitting of “After reaction 180° C”.

	Mn-O			Mn-Mn				
Sample	R (Å)	N	σ^2 (Å^2)	R (Å)	N	σ^2 (Å^2)	E_0 (eV)	R-factor
After H_2 400°C	2.19 ± 0.03	6	0.015	3.16 ± 0.02	12	0.014	-0.7 ± 0.9	0.013
After Reaction 180°C	2.18 ± 0.04	6	0.011	3.15 ± 0.01	12	0.013	-2.0 ± 0.8	0.009

Table S2 – EXAFS (Mn K-edge) fitting results of Mn(0.3)MoS_2 after H_2 pretreatment at 400 °C and after reaction at 180 °C

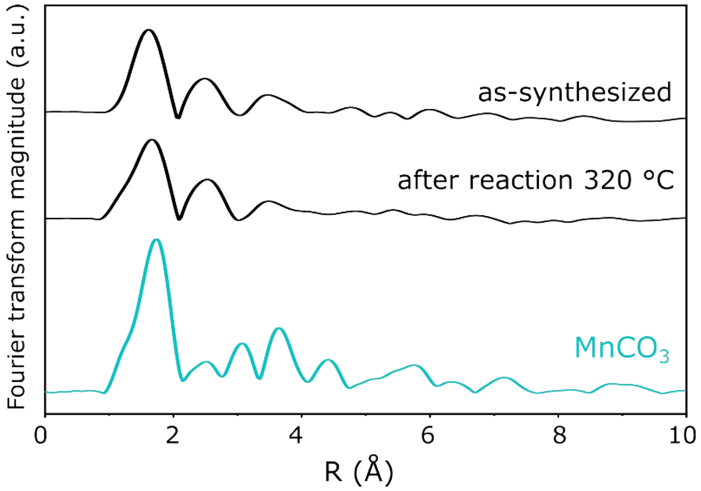

Figure S7 - EXAFS spectra of the Mn K-edge from Mn(0.3)-MoS_2 after synthesis and catalytic reaction at 320 ° C, compared to a $MnCO_3$ reference samples.

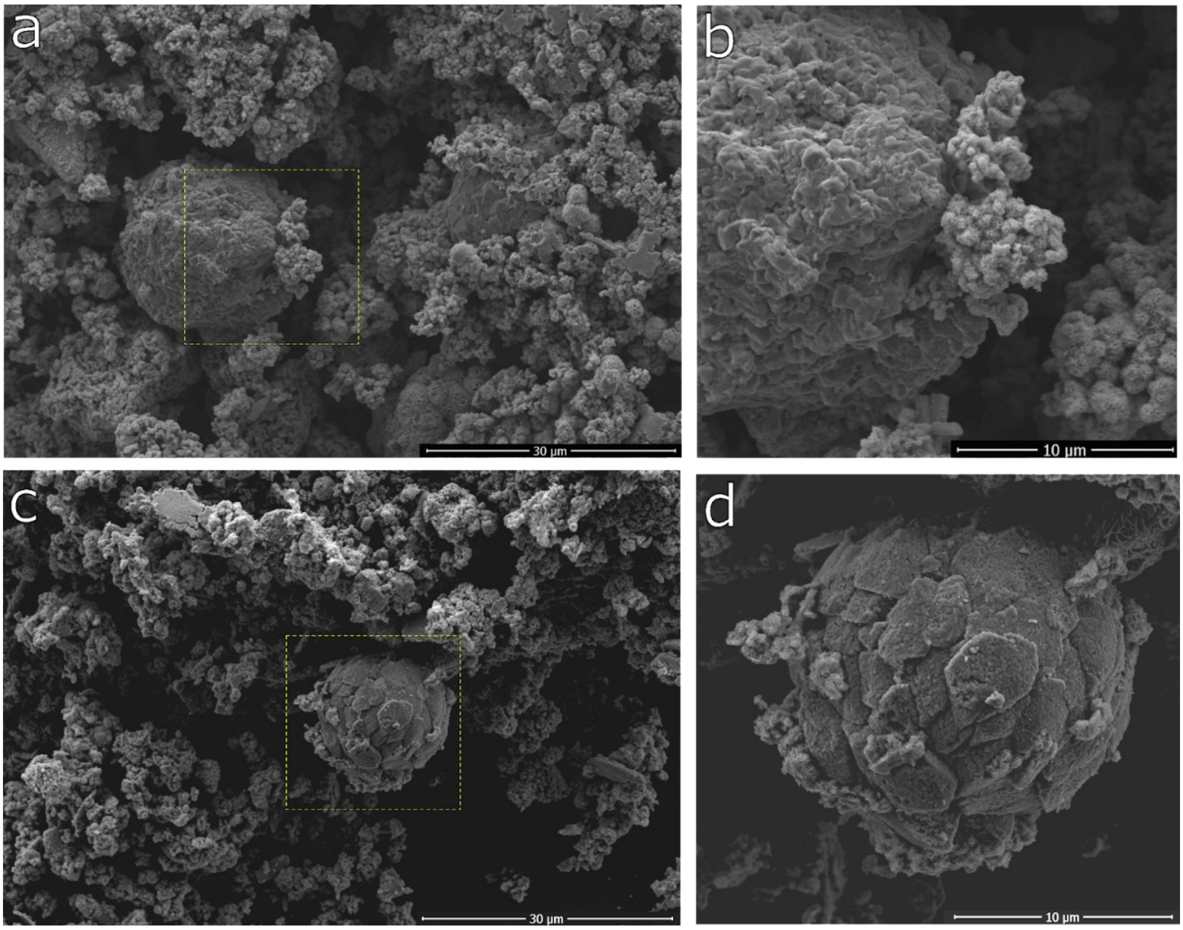

Figure S8 - Additional SEM micrographs of a,b) as-synthesized Mn(0.3)-MoS_2 and c,d) Mn(0.3)-MoS_2 after H_2 treatment and catalytic reaction at 180 °C

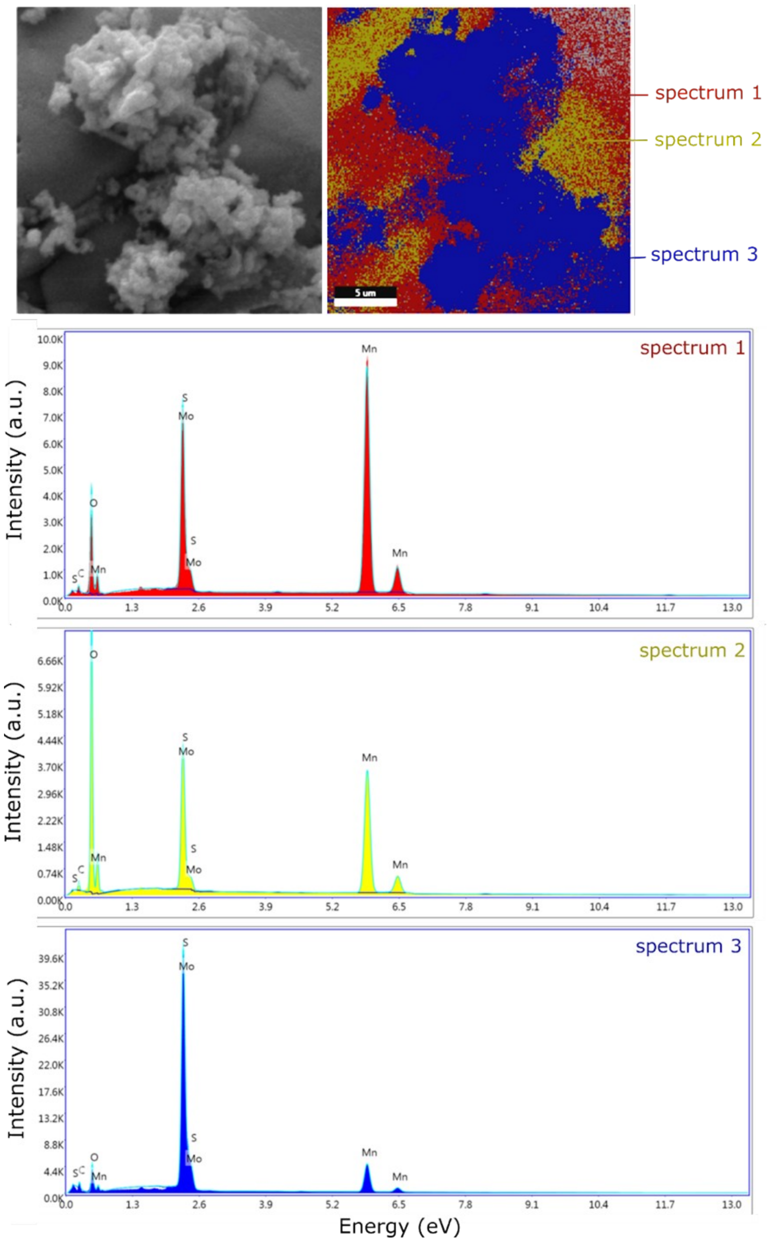

Figure S9 - SEM micrograph of the region analyzed by EDX, alongside the EDX mapping and the related spectra

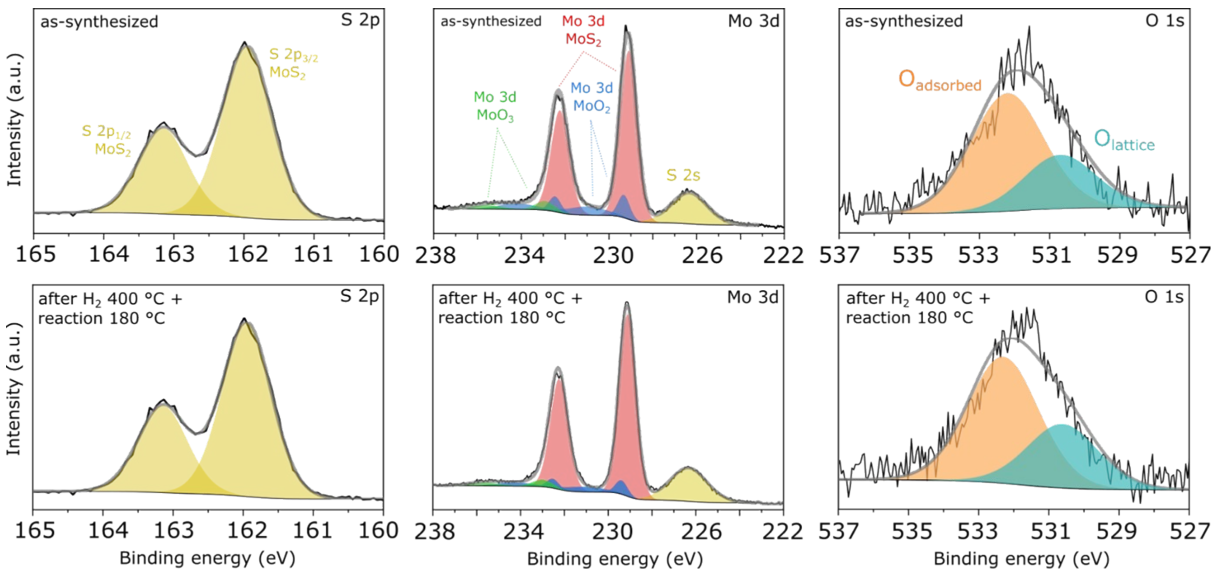

Figure S10 - NAP-XPS spectra of as-synthesized and spent Mn(0.3)-MoS_2 catalysts showing the regions S 2p, Mo 3d and O 1s

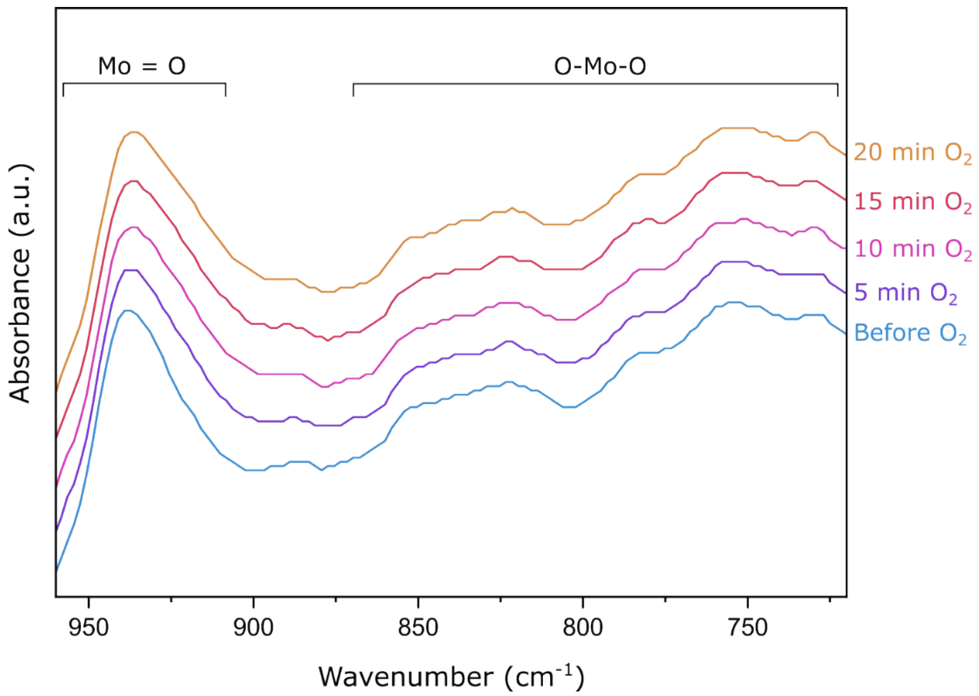

Figure S11 - DRIFTS spectra of Mn(0.3)-MoS_2 during O_2 chemisorption. The experiment was carried out using a BrukerVertex 70 spectrometer (liquid N_2-cooled MCT detector). The cell inlet was connected to mass flow controllers for O_2, H_2 and Ar. The powder sample was placed in a crucible located inside the cell. After purging with Ar, the cell was heated up to 400 °C and kept for 3h under H_2 flow. Finally, the cell was cooled to room temperature under Ar flow and O_2 flow was introduced while IR spectra were periodically recorded.

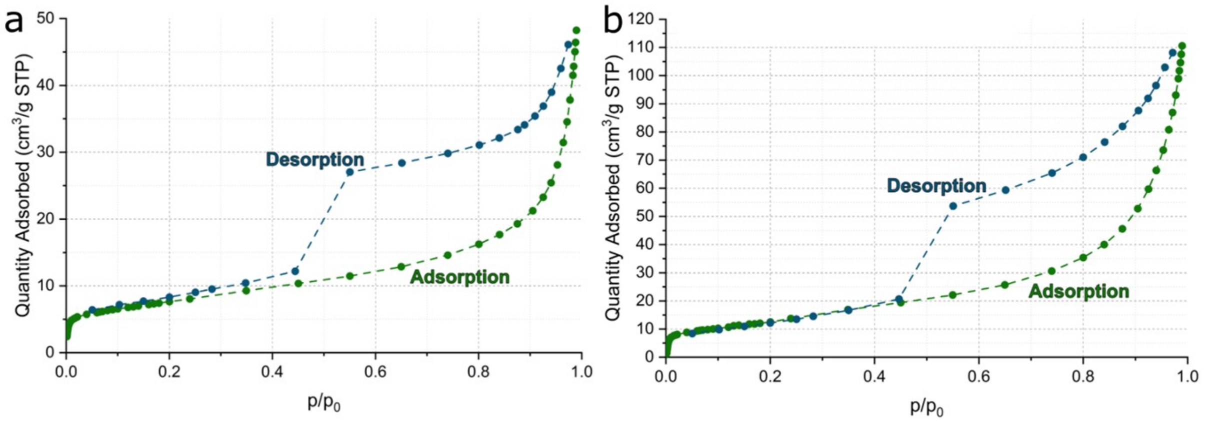

Figure S12 - N_2 adsorption-desorption isotherms at -196 °C of a) pure MoS_2 and b) Mn(0.3)-MoS_2. Analysis was conducted with a Micromeritics ASAP 2020, after degassed at 350 °C for 8 h under vacuum. Specific surface areas were calculated using the Brunauer-Emmett-Teller (BET) method, with the respective values shown in Table S3.

Catalyst	CH_3OH sel. (mol%)	CO_2 conv. (mol%)	T (°C)	P (bar)	GHSV (mL $g_{cat}^{-1}h^{-1}$)	Cat. mass (g)	SA (m^2g^{-1})	H_2:CO_2 ratio	Ref.
MoS_2	0	2.0	180	20	300	1.00	25	3:1	This work
Mn(0.3)-MoS_2	64	2.8	180	20	300	1.00	41	3:1	This work
FL-MoS_2	94	12.5	180	50	3000	0.20	130	3:1	[4]
Cu/MoS_2@SiO_2	93	3.0	180	50	8000	0.15	98	4:1	[5]
MoS_2/ZnS	88	2.1	180	50	6000	0.20	86	4:1	[6]
MoS_2/$Ni_{0.2}$	84	1.0	260	50	12000	0.20	41	3:1	[7]
MoS_2/$Co_{0.2}$	74	0.7	260	50	12000	0.20	n.a.	3:1	[7]

Table S3 – Comparison of catalytic performance of materials presented in this work with previously reported MoS_2-based catalysts, in terms of CH_3OH selectivity, CO_2 conversion, reaction temperature, pressure, gas hourly space velocity, catalyst mass, surface area and H_2:CO_2 ratio

References

1 R.E.G. Pacalo, E.K. Graham. Pressure and temperature dependence of the elastic properties of synthetic MnO, *Physics and Chemistry of Minerals* 18, 69-80 (1991)

2 R.W.G. Wyckoff. Crystal Structures 1 (1963) 7-83, Second edition. Interscience Publishers, New York (AMCSD code 0011221)

3 R.W.G. Wyckoff, Crystal Structures 1 (1963) 280-281, Second edition. Interscience Publishers, New York (AMCSD code 0011827)

4 J. Hu, *et al*. Sulfur vacancy-rich MoS2 as a catalyst for the hydrogenation of CO2 to methanol. *Nature Catalysis* 4, 242-250 (2021)

5 S. Zhou *et al*. Strained few-layer MoS2 with atomic copper and selectively exposed in-plane sulfur vacancies for CO2 hydrogenation to methanol. *Nature Communications* 14, 5872 (2023)

6 S. Zhou, H.C. Zeng. Boxlike Assemblages of Few-Layer MoS2 Nanosheets with Edge Blockage for High-Efficiency Hydrogenation of CO2 to Methanol. *ACS Catalysis* 12, 16, 9872–9886 (2022)

7 Y. Yuan, *et al.* Performance Exploration of Ni-Doped MoS2 in CO2 Hydrogenation to Methanol. *Molecules* 28, 15, 5796 (2023)
